# Nationwide survey on HER2 and PD-L1 testing practices in gastric cancer across Japan

**DOI:** 10.1007/s10120-024-01571-w

**Published:** 2024-12-10

**Authors:** Hiroyuki Abe, Takeshi Kuwata, Ryoji Kushima, Tetsuo Ushiku

**Affiliations:** 1 Japanese Gastric Cancer Association, Kyoto, Japan; 2 Department of Pathology, Graduate School of Medicine, The University of Tokyo, 7-3-1 Hongo, Bunkyo-ku, Tokyo, 113-0033 Japan; 3 Department of Genetic Medicine and Services, National Cancer Center Hospital East, Kashiwa, Japan; 4 Department of Pathology, Shiga University of Medical Science, Otsu, Japan

**Keywords:** Gastric cancer, Biomarker testing, HER2, PD-L1, Questionnaire survey

## Abstract

**Background:**

Since HER2 and PD-L1 testing are key to selecting drugs for first-line treatments in advanced gastric cancer, evaluating differences in these tests among institutions is necessary to standardize treatment.

**Methods:**

A questionnaire survey was conducted targeting institutions certified by the Japanese Gastric Cancer Association.

**Results:**

Responses were obtained from 155 institutions. Most institutions performed HER2 testing in-house, while PD-L1 tests were largely outsourced. HER2 scores and PD-L1 CPS rates showed greater variability across institutions than anticipated. In the pre-analytic phase, 10% neutral buffered formalin was commonly used, with fixation practices generally following guidelines. Overall, the impact of fixation-related factors was limited, but in surgical specimens, longer fixation was associated with a higher proportion of score 0/1+ and a lower proportion of score 3+. When examining HER2 scores by institution, if a particular score had a high (or low) frequency in biopsy, the same trend was also seen in surgical specimens.

**Conclusions:**

These findings suggest that not only factors related to specimen preparation, but also biases in evaluation criteria among pathologists may contribute to the significant variability among institutions. Standardization of pre- and post-analytic phases, coupled with appropriate training, is essential to achieve consistent gastric cancer therapy.

**Supplementary Information:**

The online version contains supplementary material available at 10.1007/s10120-024-01571-w.

## Introduction

The major target molecule in unresectable or metastatic gastric cancer are HER2 and PD-L1, both requiring immunohistochemical tests for appropriate patient selection [1, 2]. HER2 immunohistochemistry is assessed by the membranous staining of cancer cells, with specimens scored as 0, 1+, 2+, or 3+ based on the intensity and proportion of stained cells. PD-L1 expression is evaluated using the combined positive score (CPS), calculated by dividing the number of PD-L1 positive cancer cells and immune cells by the total number of cancer cells [3, 4]. In the Checkmate 649 trial, which served as the basis for the initial first-line approval of nivolumab, CPS cut-offs of 1 and 5 were used [5].

Interobserver or interlaboratory discrepancies have been reported in both HER2 and PD-L1 testing [6–9]. Additionally, pre-analytic factors, such as cold ischemic time, fixation duration, and formalin concentration, influence the results [3, 10]. Inaccurate evaluation may disadvantage patients by preventing access to appropriate therapies. To address these issues, we conducted a nationwide questionnaire survey on HER2 and PD-L1 testing in Japan, aiming to contribute to the standardization of gastric cancer treatment.

## Materials and methods

The questionnaire survey targeted 306 institutions certified by Japanese Gastric Cancer Association (JGCA), with differing standards required for institutions classified A (*n* = 127) and B (*n* = 179) (e.g., number of gastric cancer cases treated annually, number of specialists involved; Supplementary Table 1). The questionnaire comprised 10 questions designed to assess current practices in HER2 and PD-L1 testing. The questionnaire are as follows:

1. Which tests are performed before initiating chemotherapy for gastric cancer?
2. Where is the HER2 test conducted, and how many HER2 tests are performed annually?
3. What diagnostic kit is used for HER2 tests?
4. Are scores of 0 and 1+ reported separately, or are they combined into one category (e.g., 0/1+ or negative)?
5. How many cases were scored as 0, 1+, 2+, and 3+ in biopsy and surgical specimen, respectively?
6. Where is the PD-L1 test conducted, and how many PD-L1 tests are performed annually?
7. How many cases are evaluated as CPS < 1, 1 ≤ CPS < 5, and CPS ≥ 5?
8. What type of fixative is used for biopsy and surgical specimens, respectively?
9. What is the time interval between sampling and fixation (cold ischemic time)?
10. What is the fixation duration for biopsy and surgical specimens?

The questionnaire survey covered performance data from April 2022 to March 2023. Responses were collected online via Google Forms, with the survey link distributed by e-mail. The survey was conducted following approval from the Board of Directors of JGCA. As the survey did not involve patient data or samples, approval from an institutional review board was waived.

Statistical analyses were performed with Welch’s t test for continuous variables. For categorical data, Fisher’s exact test was used, and correlation coefficient was calculated.

## Results

### Survey results on testing performance and methods

**Fig. 1:**
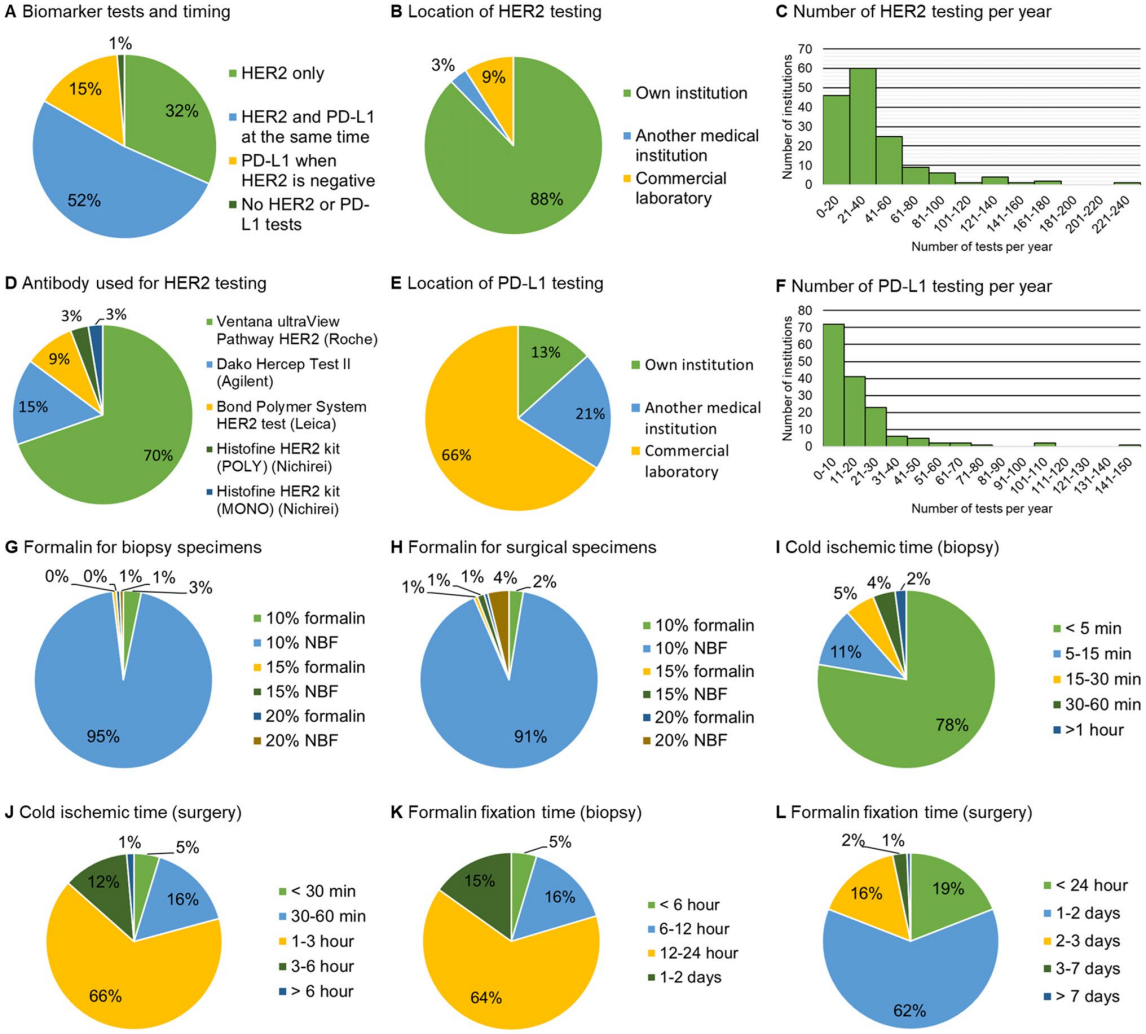
Summary of survey results. **A** Implementation and timing of HER2 and PD-L1 testing. **B** Location where HER2 testing is conducted. **C** The number of HER2 test per year in each institution. **D** HER2 detection kit used in each institution. **E** Location where PD-L1 test is performed. **F** The number of PD-L1 test per year. **G** Types of fixative used for biopsy specimen. **H** Types of fixative used for surgically resected specimen. **I** Time between endoscopic biopsy and fixation. **J** Time between surgical removal and fixation. **K** Formalin fixation duration for biopsy specimen. **L** Formalin fixation duration for surgically resected specimen. Most institutions follow the guidelines for pre-analytic practices; however, some facilities deviate from recommendations, such as having excessively long fixation times. *NBF*, neutral buffered formalin

Survey responses were received from 155 out of 306 institutions (51%) (A, 60% [76/127]; B, 44% [79/179]). Of these, 80 institutions (52%) performed both HER2 and PD-L1 testing simultaneously before chemotherapy. Another 49 institutions (32%) performed only the HER2 test, while 24 (15%) conducted the PD-L1 test only for HER2-negative cases (Fig. 1A).

Most institutions (88%) performed the HER2 test in-house, while the rest outsourced the test to other medical facilities or commercial laboratories (Fig. 1B). The annual number of HER2 tests ranged from 0 to 227, with a median of 31 (Fig. 1C). The most popular diagnostic kit was the Ventana ultraView Pathway HER2 (Roche), used by 108 institutions (70%); followed by Dako HercepTest II (Agilent) in 24 institutions (15%) and others (< 10%) (Fig. 1D).

Regarding PD-L1 testing, only 20 institutions (13%) conducted the test in-house, while the majority outsourced it (Fig. 1E). The number of PD-L1 tests per year ranged from 0 to 149, with a median of 12, significantly lower than the number of HER2 tests (P < 0.001) (Fig. 1F).

Survey results on the fixation-related factors are summarized in Fig. 1G–L. For biopsy samples, 147 (95%) institutions used 10% neutral buffered formalin (NBF). The time between biopsy and fixation was less than 5 min in 115 (78%) institutions, although it exceeded 30 min in 9 (6%) institutions. Fixation length was under 24 h in 129 (85%) institutions. For surgical specimens, 10% NBF was used in 141 (91%) institutions. The time between resection and fixation was less than 3 h in 129 (87%) institutions, and fixation length was under 2 days in 123 (81%) institutions.

### Variation of HER2 scores distribution

**Fig. 2:**
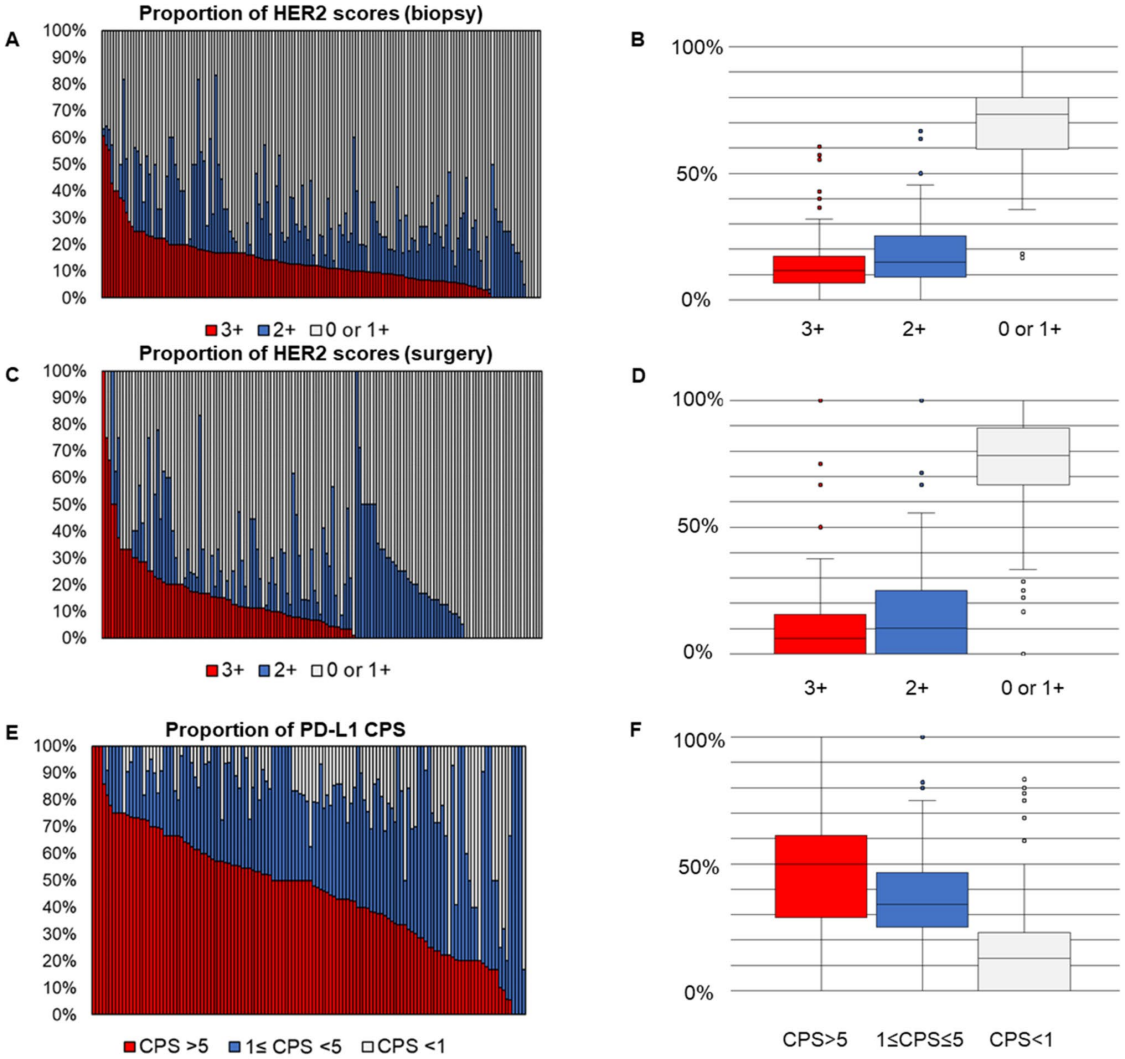
Distribution of HER2 scores and PD-L1 scores. Proportions of HER2 scores in biopsy specimen (**A**, **B**) and surgical specimen (**C**, **D**), and PD-L1 CPS (**E**, **F**). Figures **A**, **C** and **E** show the proportions of each score in each institution using 100% stacked bar charts. Figures **B**, **D**, and **F** show the percentage distribution of each score using box plots

The study analyzed 5,129 biopsy cases and 2,514 surgical cases for HER2 expression, with biopsy samples showing 69% as 0 or 1+, 17% as 2+, and 14% as 3+, while surgical samples showed 77% as 0 or 1+, 14% as 2+, and 9% as 3+. The distribution of HER2 scores varied significantly among institutions (Fig. 2A–D). The proportions of score 3+ ranged from 0 to 61% (mean 13% ± 11%) in biopsy specimens, and from 0 to 100% (mean 10% ± 15%) in surgical specimens. The proportion of negative results (score 0 or 1 +) also varied widely, ranging from 17 to100% (mean 69% ± 17%) in biopsy specimens, and from 0 to 100% (mean of 74% ± 23%) in surgical specimens. The proportions of scores 0 and 1+ were also highly variable (Supplementary Fig. 1). The high variability persisted even among institutions performing more than 20 tests annually (Supplementary Fig. 2).

### Variation in PD-L1 positivity rates among institutions

PD-L1 expression data were available for 2,533 cases, distributed as follows: CPS < 1 in 480 cases (19%), CPS between 1 and 5 in 855 cases (34%), and CPS ≥ 5 in 1,198 cases (47%). PD-L1 test results also showed significant variability across institutions (Fig. 2E–F). The proportion of cases with CPS ≥ 5 ranged from 0 to 100% (mean 46% ± 22%). For CPS < 1, it ranged from 0 to 83% (mean 17% ± 20%). This variability was also observed even when limited to institutions testing more than 20 cases annually (Supplementary Fig. 3).

### Correlation among factors

**Fig. 3:**
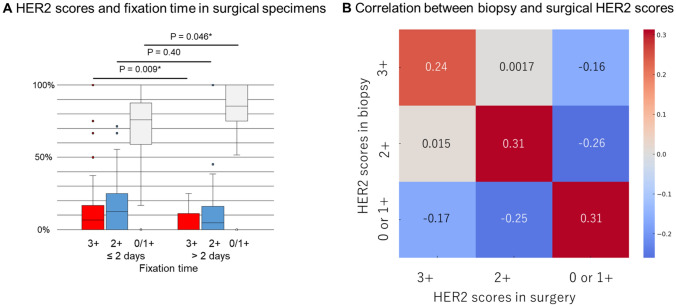
Factors showing a trend in correlation analysis. **A** Distribution of HER2 scores in surgical specimens at institutions with fixation times ≤ 2 days vs. > 2 days. The latter has a significantly lower proportion of score 3+ (*P* = 0.009) and a higher proportion of scores 0/1+ (*P* = 0.046) (Welch t-test). **B** Correlation of HER2 scores between biopsy and surgical specimens. Moderate positive correlation was observed between the same scores, indicating that institutions with a higher (or lower) proportion of a specific score in biopsy specimens tended to have a similarly higher (or lower) proportion of that score in surgical specimens

Regarding correlation between HER2 scores and fixation time in surgical specimens, longer fixation time was associated with a higher proportion of score 0/1+ (*P* = 0.046) and a lower proportion of score 3+ (*P* = 0.009) (Fig. 3A). Aside from this, there was little correlation between fixation-related factors—namely, the type of formalin, cold ischemic time, and fixation duration—and the HER2 and PD-L1 test scores (Supplementary Fig. 4).

When comparing HER2 scores between biopsy and surgical specimens at each institution, the frequency of a specific HER2 score in biopsy specimens showed a moderate positive correlation with the same score in surgical specimens (Fig. 3B). For example, institutions with a high (low) proportion of HER2-negative (0 or 1 +) results in biopsy specimens tend to also have a higher (lower) proportion of HER2-negative results in surgical specimens.

There were no significant differences in HER2 positivity rates among different HER2 detection kits (Supplementary Fig. 5). No clear trend was observed in any test results between institutions certified as A or B by the JGCA (Supplementary Fig. 6).

## Discussion

The survey revealed significant variability in the HER2 and PD-L1 tests results among Japanese institutions. Although the HER2 positivity rate in gastric cancer is generally around 20% [11–13], the survey found HER2 2+ /3+ rates ranged from 0 to 83% with mean of 31%. Similarly, PD-L1 CPS ≥ 1 frequency, typically reported as 65–80% in clinical trials [2, 14–16], varied from 17 to 100% (mean 83%). Such variability among institutions has also been noted in a study in Germany, Austria, and Switzerland [17]. A more recent study in Italy also reported wide variation in HER2 testing results among institutions [18].

Test results are influenced by pre-analytic (sample handling), analytic (immunohistochemistry procedure), and post-analytic (pathologist evaluation) phases. Given that immunohistochemistry procedures are strictly standardized, the pre- and post-analytic phases are likely the primary sources of variability in results.

Most institutions used 10% NBF, with biopsy samples promptly fixed and fixation times under 24 h. For surgically resected specimens, cold ischemic time was typically less than 3 h, and fixation time was within 2 days, as per Japanese guidelines [10]. A survey by the Japanese Association of Medical Technologists showed an increase in institutions using 10% NBF from 2015 to 2019 (from 38.5 to 80.0% in biopsy samples and from 31.6 to 72.1% in surgically resected specimens) [19], likely due to the widespread use of genomic testing and increased awareness of the guidelines for the handling of pathological specimens for genomic medicine issued by the Japanese Society of Pathology [10]. However, some institutions still employed suboptimal fixatives, cold ischemic times, or fixation lengths.

In preceding studies, cold ischemic time more than 120 min were associated with low HER2 positivity [20], and longer fixation times over weekends were associated with reduced HER2 positivity rates [21], highlighting the importance of proper sample handling. In this study, although the correlation was weak, longer fixation times in surgical specimens tended to increase HER2 score 0/1+ results. Notably, the frequency of specific HER2 scores in biopsy specimens showed a moderate positive correlation with the same scores in surgical specimens. Considering the minimal impact of fixation-related factors and differences in fixation conditions between biopsy and surgical specimens, it is suggested that post-analytic influences, in addition to pre-analytic factors, may contribute to variability. Particularly, there may be biases in evaluation criteria among pathologists. For example, in borderline cases between score 2+ and score 1+, one pathologist may tend to classify it as 2+, while another may prefer to classify it as 1+. Previous studies have shown that appropriate training can improve concordance in HER2 evaluations [22]. Implementing regular monitoring of HER2 positivity rates and training programs for institutions with unusual rates could reduce variability in HER2 and PD-L1 testing, thereby helping to standardize gastric cancer treatment.

Recently, trastuzumab deruxtecan was shown to be effective for HER2 low (2+ without gene amplification or 1 +) or ultra-low (score 0 with faint or incomplete membrane staining in less than 10% of tumor cells) breast cancer in clinical trials [23, 24]. Although the evidence of trastuzumab deruxtecan in gastric cancer is limited [25], it was already reported that evaluation of HER2-low in gastric cancer is highly variable among institutions [18]. Standardization of HER2-low evaluation is an issue for further study.

This study is limited by its focus on JGCA-certified institutions, which may not reflect broader trends. Furthermore, the response rate (51%) was relatively low. Additionally, the survey did not include in situ hybridization for HER2 in 2+ cases. At the time of this survey, only the 28–8 clone was used for PD-L1 testing in gastric cancer, but the 22C3 antibody is now also being used, necessitating further research on their usage. Finally, we did not collect separate data for weekdays and weekends. Despite these limitations, this study provides valuable insights into HER2 and PD-L1 testing in Japan, underscoring the importance for ongoing standardizing efforts to advance personalized gastric cancer treatment.

## Supplementary Information

Below is the link to the electronic supplementary material.

Supplementary file1 (PPTX 332 KB)

Supplementary file2 (DOCX 21 KB)
