# Supplementary material for: Nationwide survey on HER2 and PD-L1 testing practices in gastric cancer across Japan
Source: Gastric Cancer. 2024 Dec 10;28(2):294–300. doi: 10.1007/s10120-024-01571-w (PMC11842516; doi:10.1007/s10120-024-01571-w)
Supplement: Supplementary file 1 — Supplementary file1 (PPTX 332 KB) [file 10120_2024_1571_MOESM1_ESM.pptx]

## Slide 1
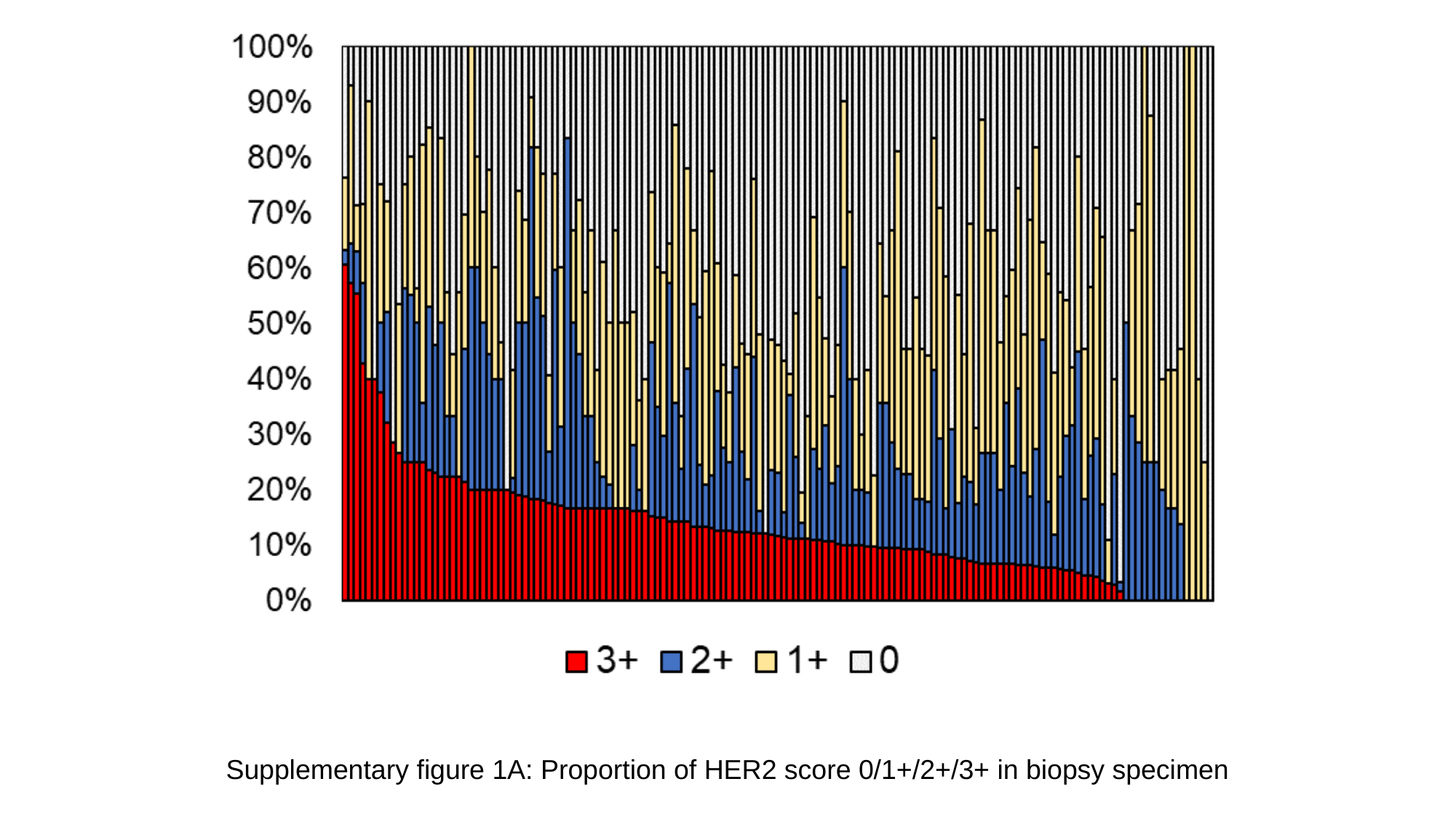

Supplementary figure 1A: Proportion of HER2 score 0/1+/2+/3+ in biopsy specimen

## Slide 2
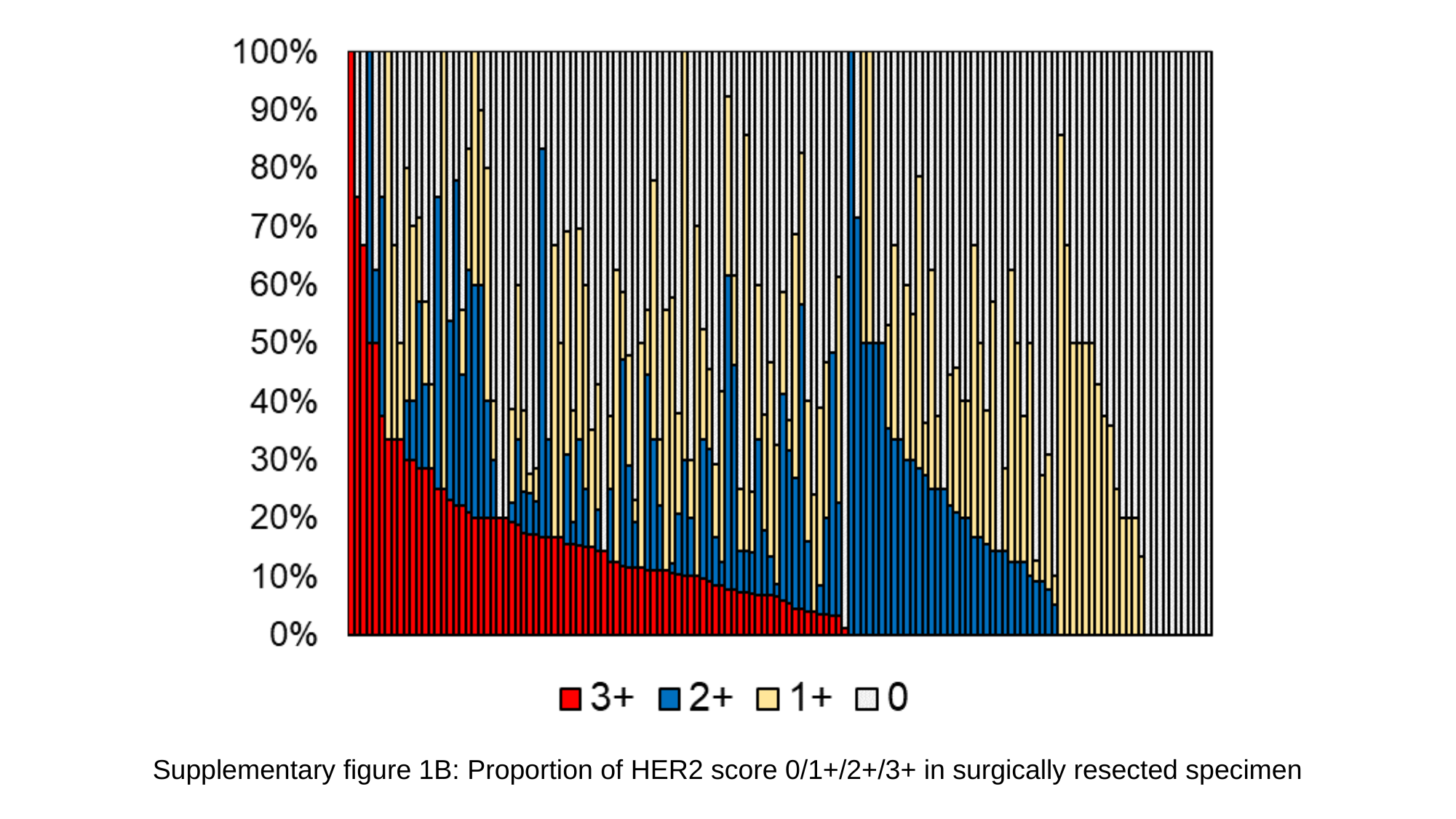

Supplementary figure 1B: Proportion of HER2 score 0/1+/2+/3+ in surgically resected specimen

## Slide 3
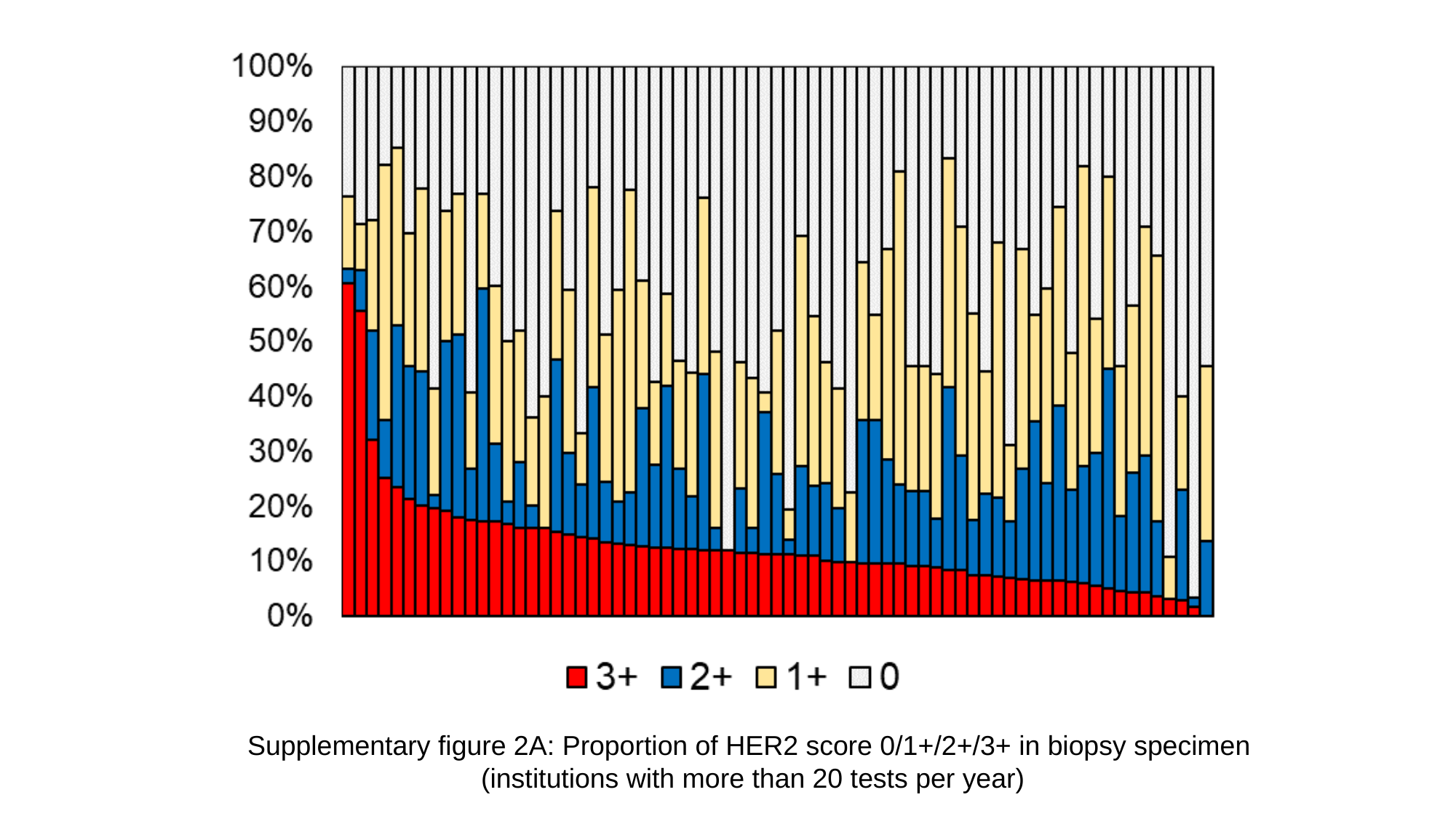

Supplementary figure 2A: Proportion of HER2 score 0/1+/2+/3+ in biopsy specimen
(institutions with more than 20 tests per year)

## Slide 4
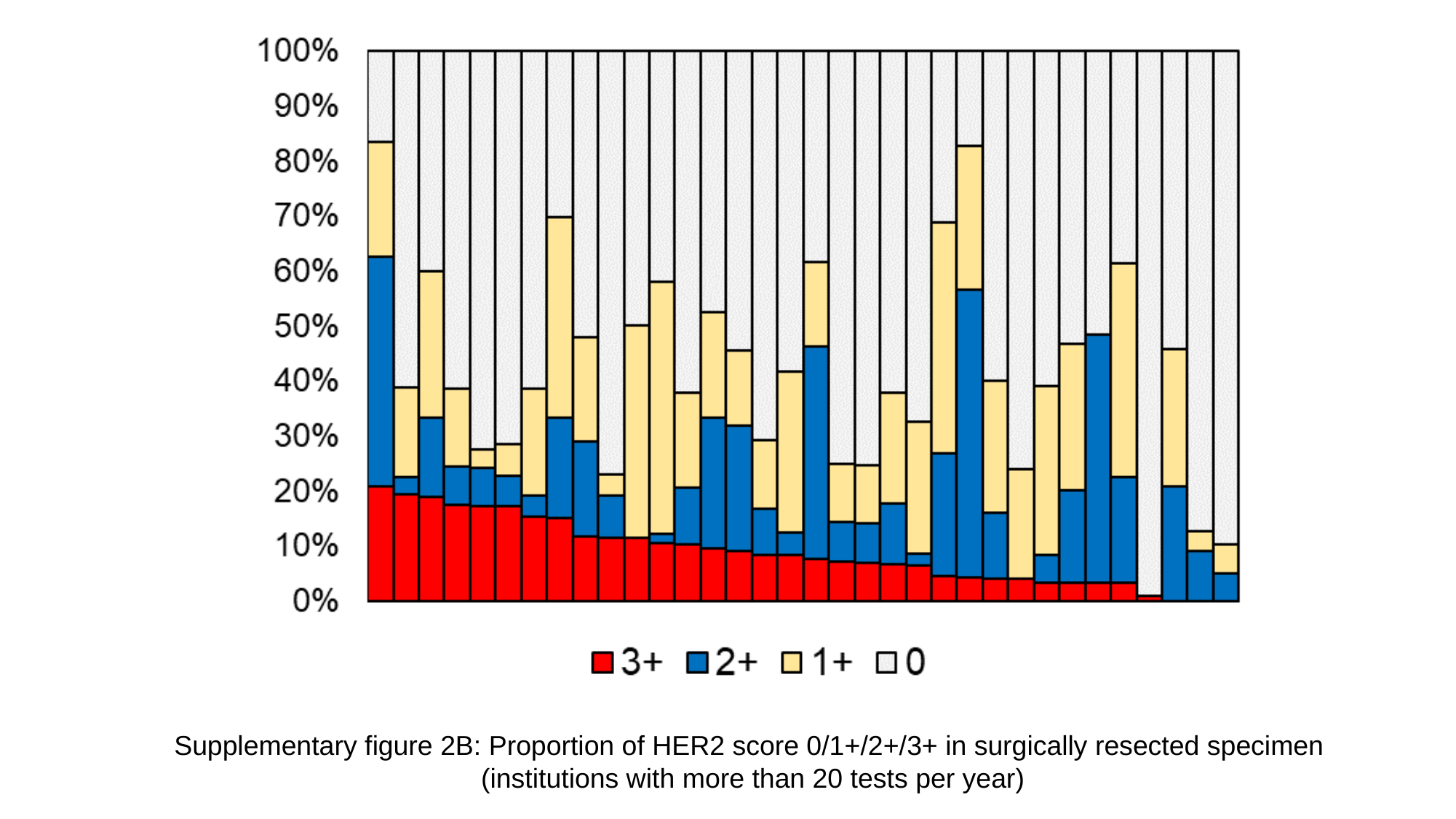

Supplementary figure 2B: Proportion of HER2 score 0/1+/2+/3+ in surgically resected specimen
(institutions with more than 20 tests per year)

## Slide 5
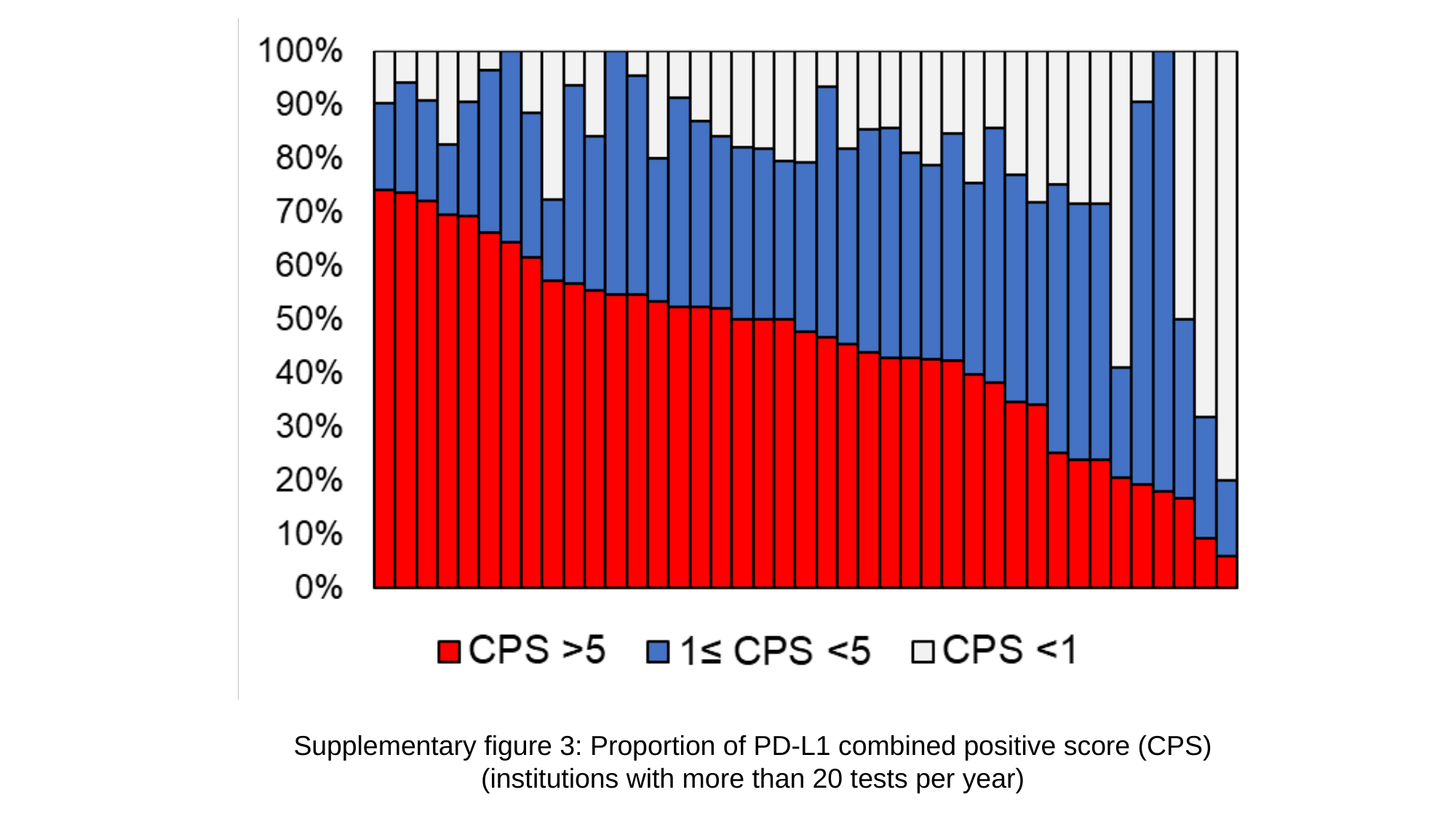

Supplementary figure 3: Proportion of PD-L1 combined positive score (CPS)
(institutions with more than 20 tests per year)

## Slide 6
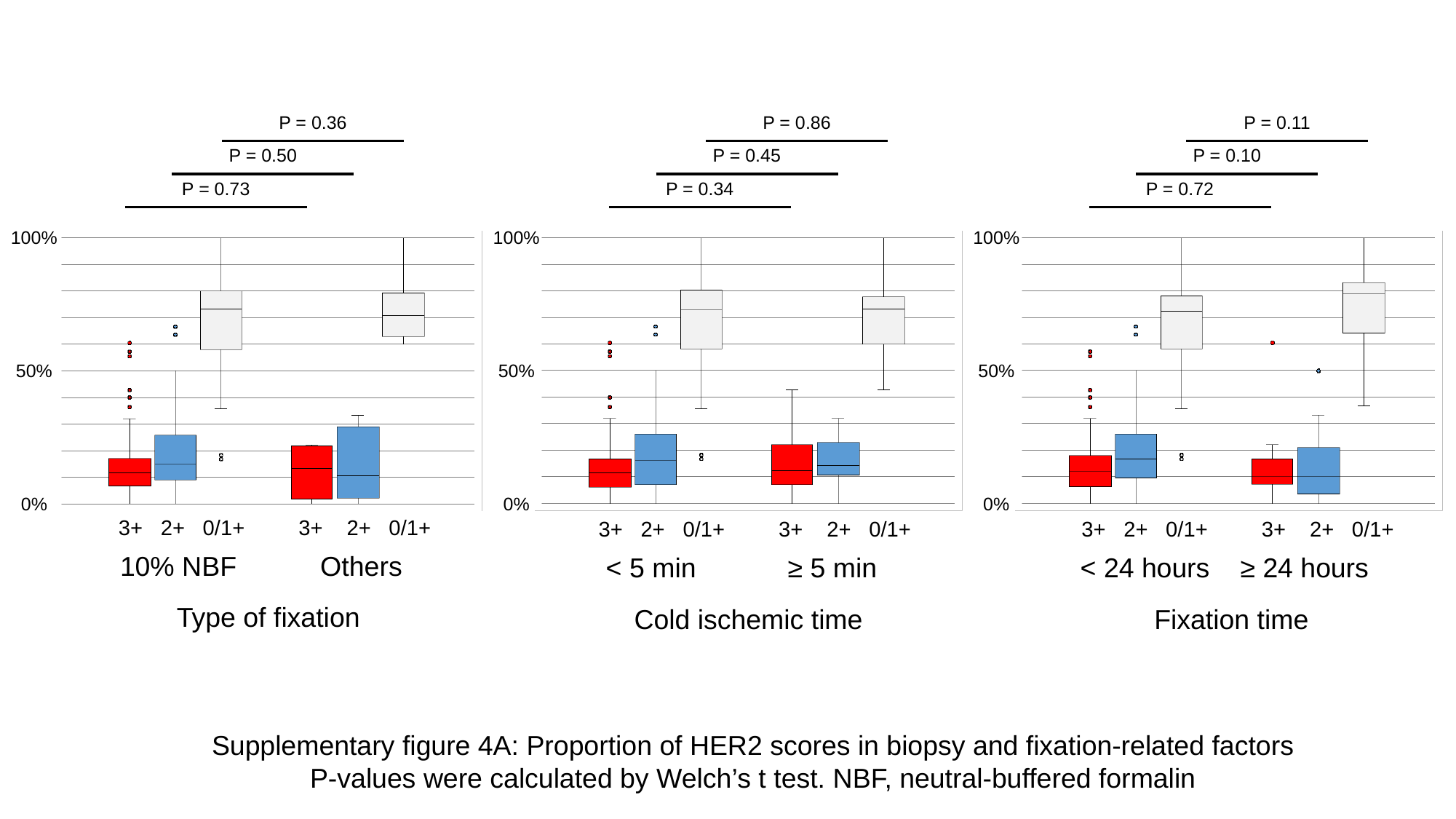

P = 0.36
P = 0.50
P = 0.73
P = 0.86
P = 0.45
P = 0.34
P = 0.11
P = 0.10
P = 0.72
100%
100%
100%
50%
50%
50%
0%
0%
0%
3+ 2+ 0/1+ 3+ 2+ 0/1+
3+ 2+ 0/1+ 3+ 2+ 0/1+
3+ 2+ 0/1+ 3+ 2+ 0/1+
10% NBF Others
< 5 min ≥ 5 min
< 24 hours ≥ 24 hours
Type of fixation
Cold ischemic time
Fixation time
Supplementary figure 4A: Proportion of HER2 scores in biopsy and fixation-related factors
P-values were calculated by Welch’s t test. NBF, neutral-buffered formalin

## Slide 7
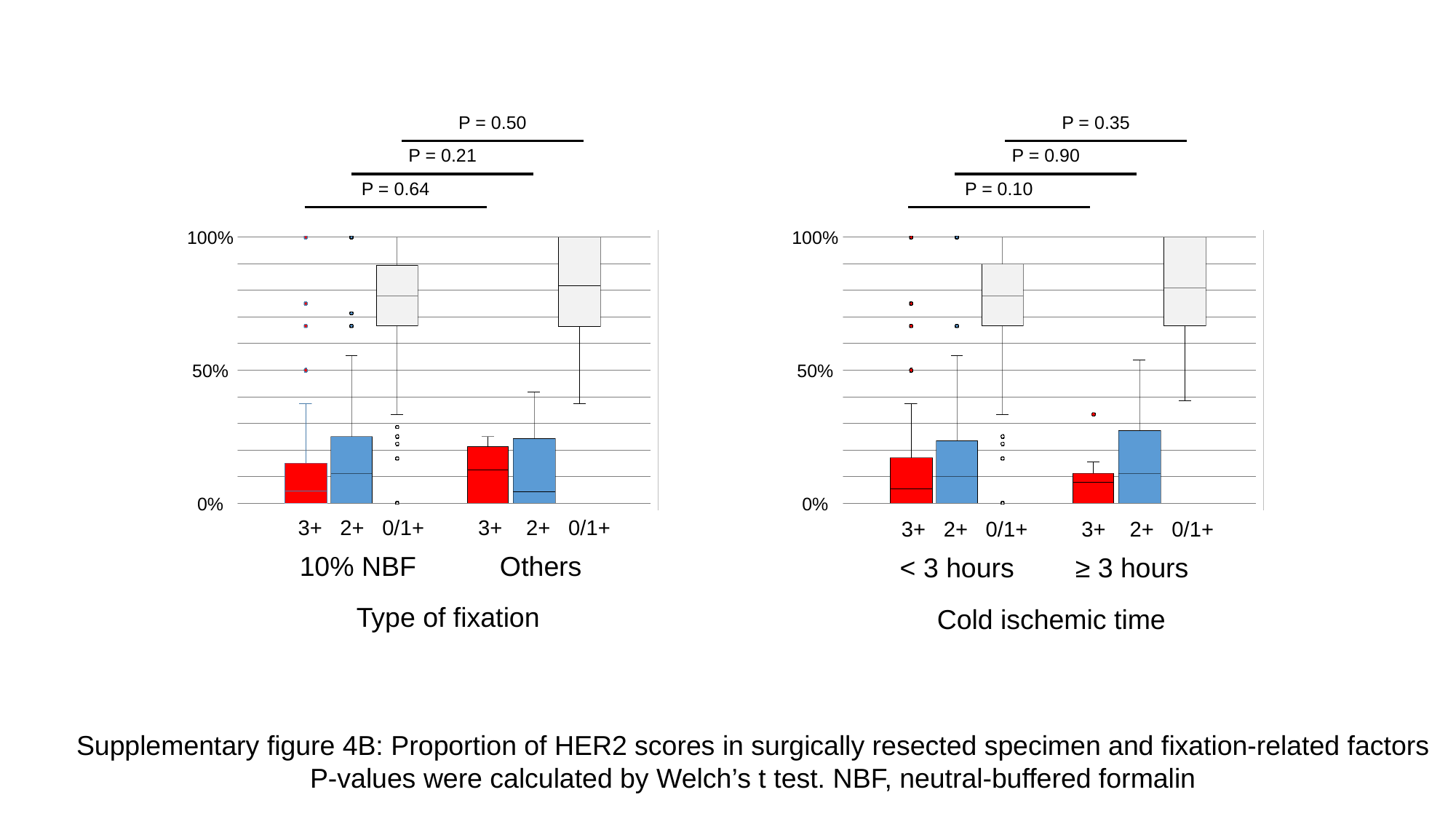

P = 0.50
P = 0.21
P = 0.64
P = 0.35
P = 0.90
P = 0.10
100%
100%
50%
50%
0%
0%
3+ 2+ 0/1+ 3+ 2+ 0/1+
3+ 2+ 0/1+ 3+ 2+ 0/1+
10% NBF Others
< 3 hours ≥ 3 hours
Type of fixation
Cold ischemic time
Supplementary figure 4B: Proportion of HER2 scores in surgically resected specimen and fixation-related factors
P-values were calculated by Welch’s t test. NBF, neutral-buffered formalin

## Slide 8
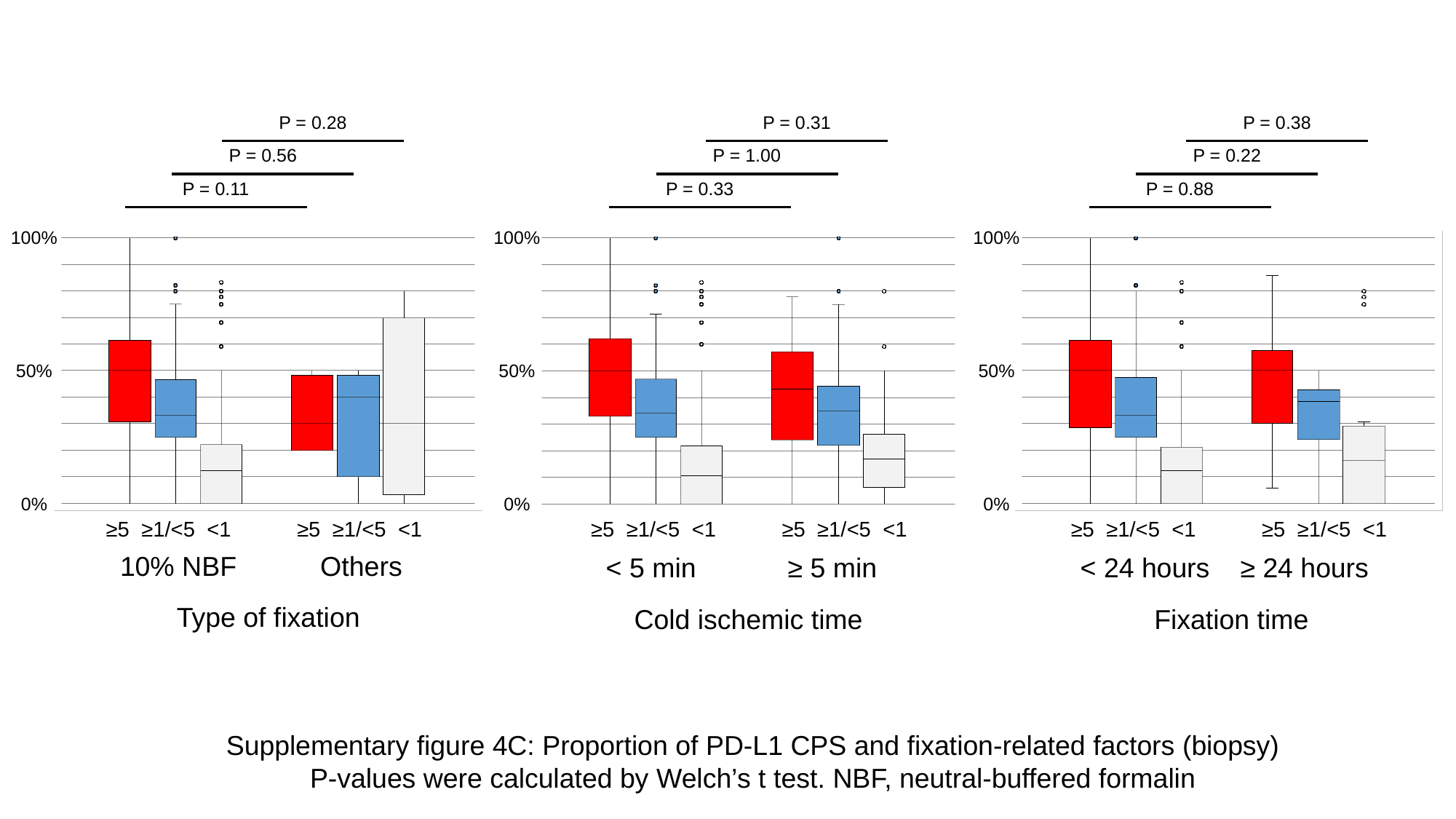

P = 0.28
P = 0.56
P = 0.11
P = 0.31
P = 1.00
P = 0.33
P = 0.38
P = 0.22
P = 0.88
100%
100%
100%
50%
50%
50%
0%
0%
0%
≥5 ≥1/<5 <1 ≥5 ≥1/<5 <1
≥5 ≥1/<5 <1 ≥5 ≥1/<5 <1
≥5 ≥1/<5 <1 ≥5 ≥1/<5 <1
10% NBF Others
< 5 min ≥ 5 min
< 24 hours ≥ 24 hours
Type of fixation
Cold ischemic time
Fixation time
Supplementary figure 4C: Proportion of PD-L1 CPS and fixation-related factors (biopsy)
P-values were calculated by Welch’s t test. NBF, neutral-buffered formalin

## Slide 9
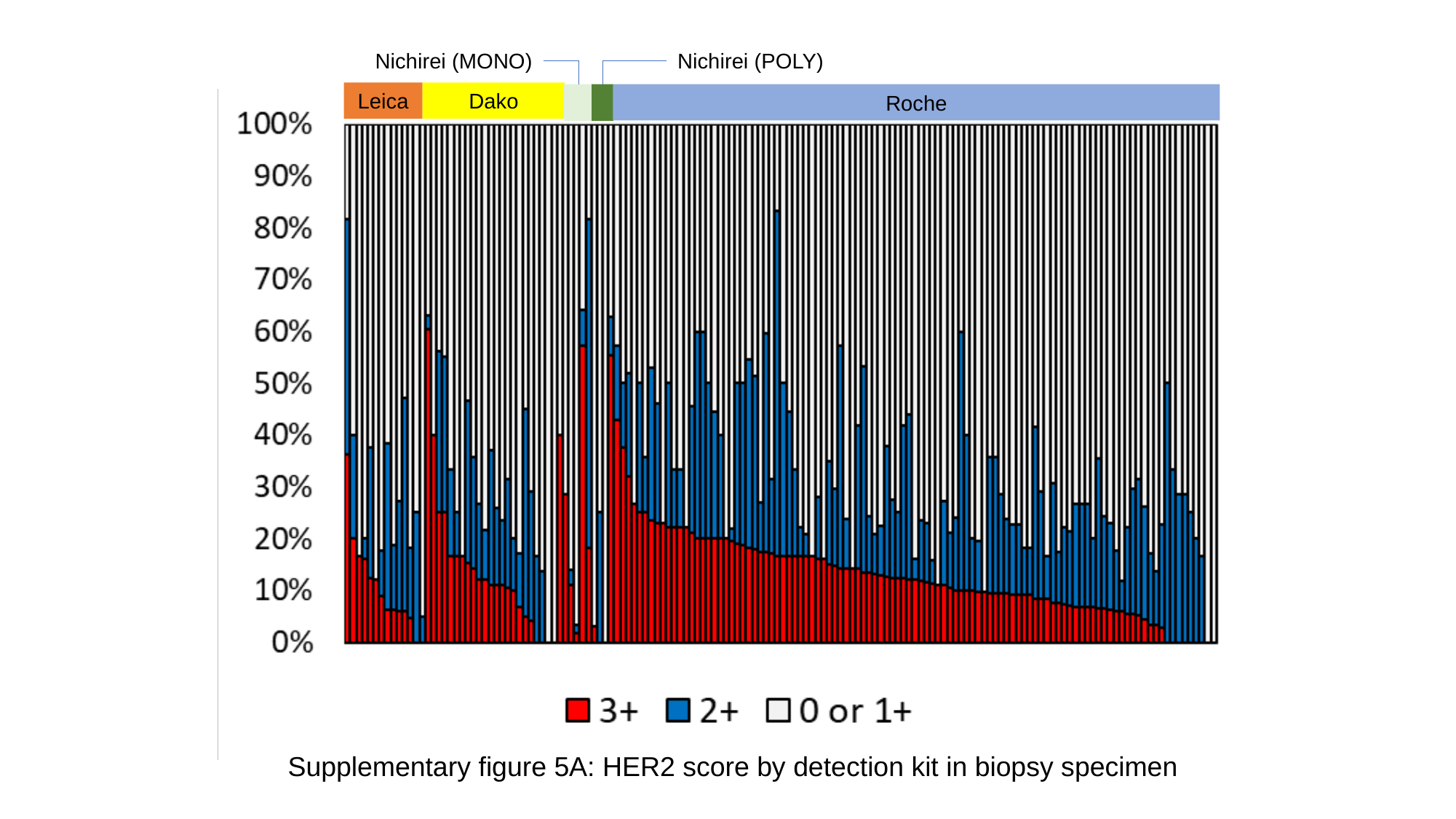

Nichirei (MONO)
Nichirei (POLY)
Leica
Dako
Roche
Supplementary figure 5A: HER2 score by detection kit in biopsy specimen

## Slide 10
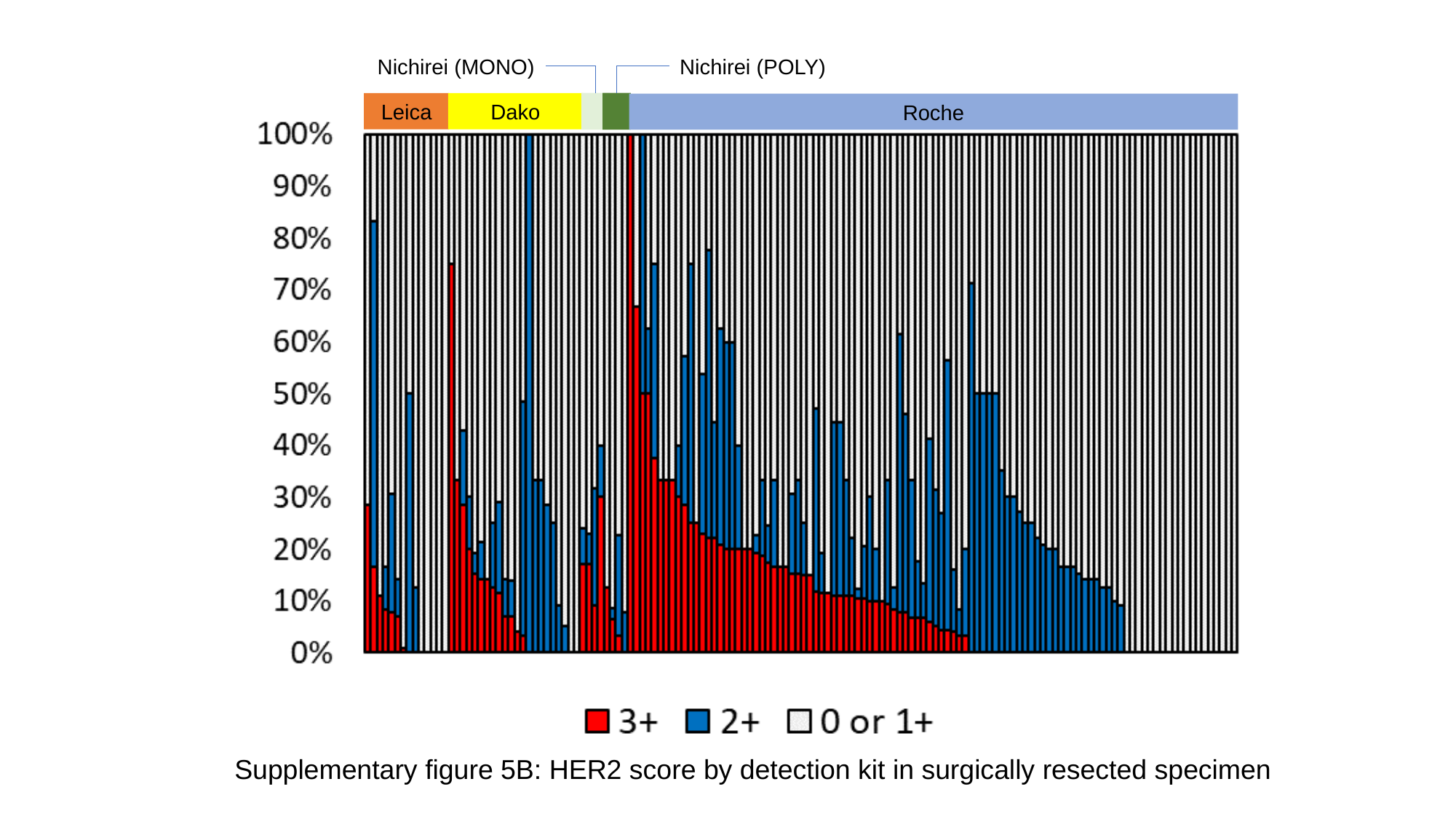

Nichirei (MONO)
Nichirei (POLY)
Dako
Leica
Roche
Supplementary figure 5B: HER2 score by detection kit in surgically resected specimen

## Slide 11
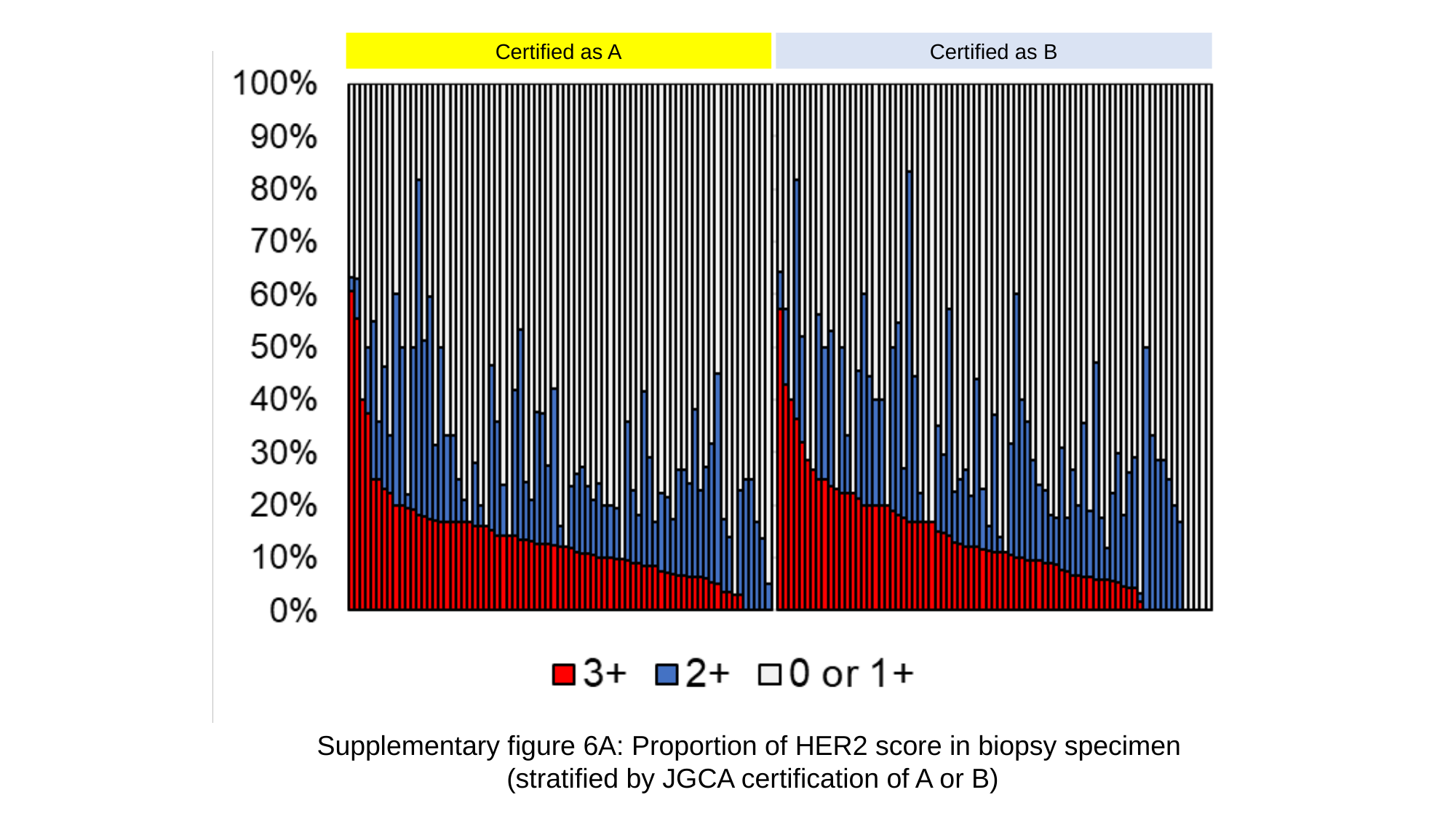

Certified as A
Certified as B
Supplementary figure 6A: Proportion of HER2 score in biopsy specimen
(stratified by JGCA certification of A or B)

## Slide 12
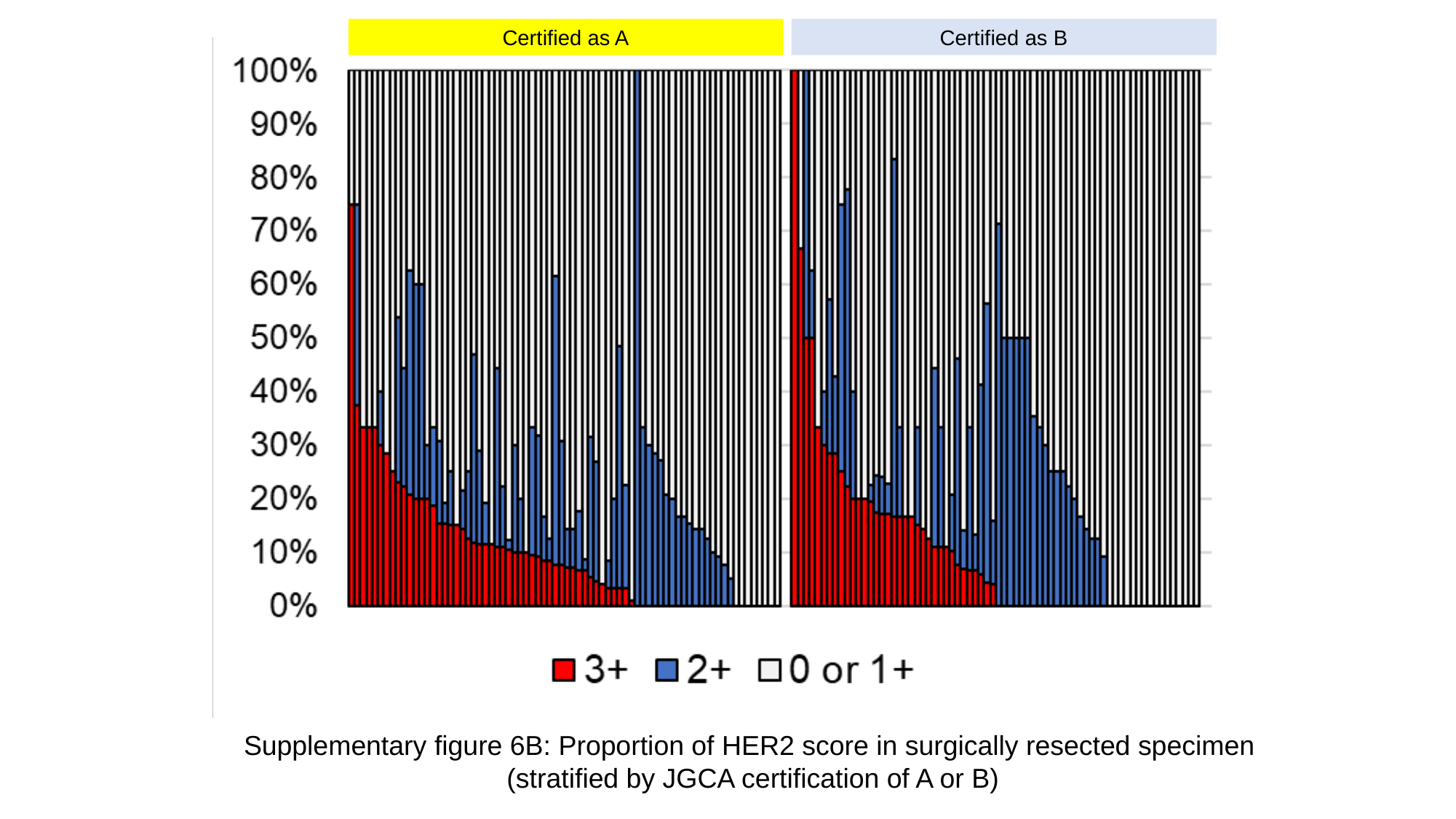

Certified as B
Certified as A
Supplementary figure 6B: Proportion of HER2 score in surgically resected specimen
(stratified by JGCA certification of A or B)

## Slide 13
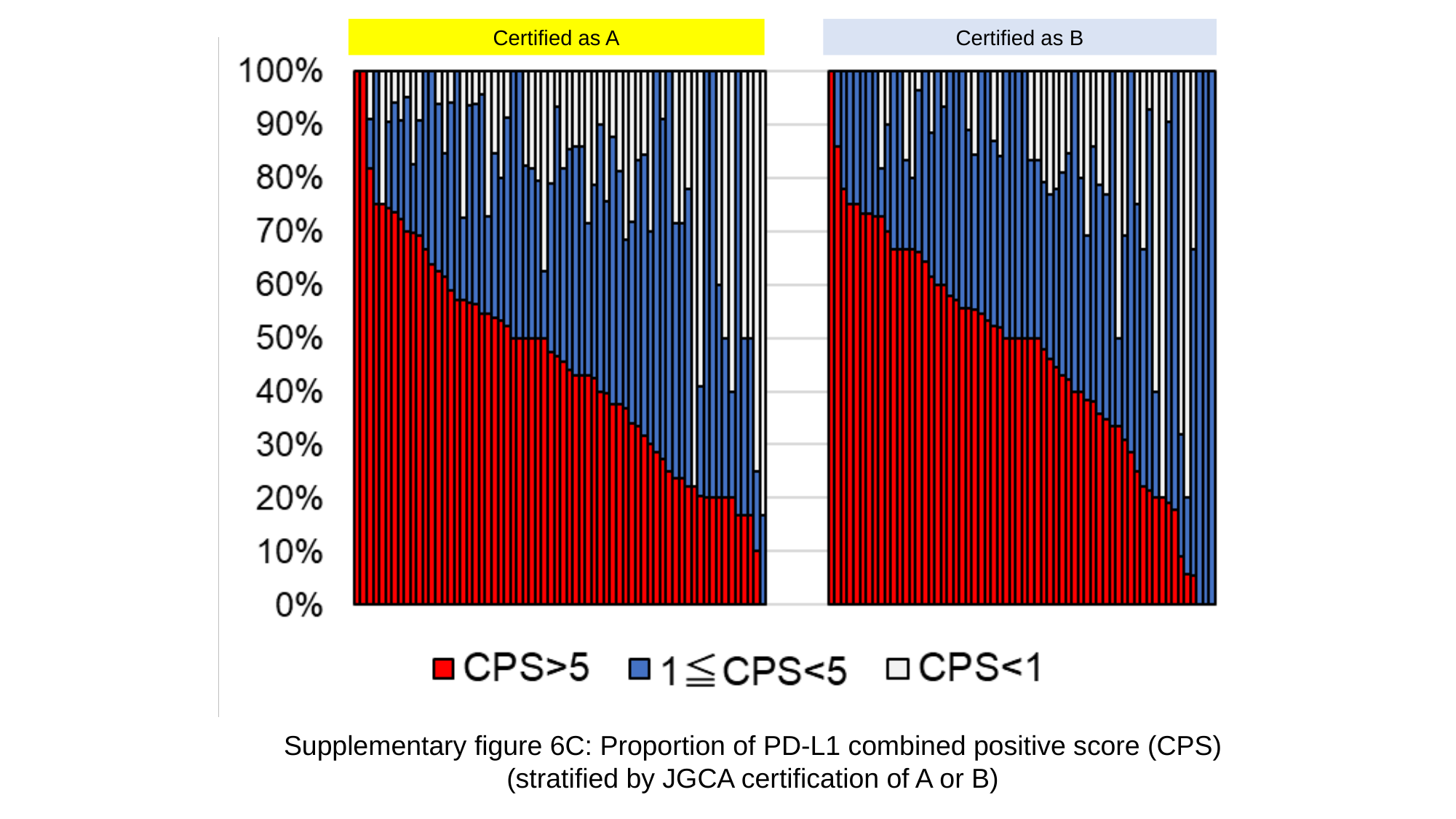

Certified as B
Certified as A
Supplementary figure 6C: Proportion of PD-L1 combined positive score (CPS)
(stratified by JGCA certification of A or B)
