# Supplementary material for: Nationwide survey on HER2 and PD-L1 testing practices in gastric cancer across Japan
Source: Gastric Cancer. 2024 Dec 10;28(2):294–300. doi: 10.1007/s10120-024-01571-w (PMC11842516; doi:10.1007/s10120-024-01571-w)
Supplement: Supplementary file 2 — Supplementary file2 (DOCX 21 KB) [file 10120_2024_1571_MOESM2_ESM.docx]

Supplementary table 1. Requirements for Certification Facilities A and B by Japanese Gastric Cancer Association

| Items | A | B |
| --- | --- | --- |
| Full-time endoscopists | ≥2 | ≥1 |
| Full-time gastrointestinal surgeons | ≥2 | ≥1 |
| Full-time endoscopic surgeons | ≥1 | No requirements |
| Full-time medical oncologists | ≥1 | No requirements |
| Full-time pathologists and cytologists | ≥1 | Part-time pathologists are acceptable |
| Participation in the Annual Meeting of JGCA | ≥6 times in three years | ≥1 time in three years |
| Presentation in the Annual Meeting of JGCA | ≥6 times in three years | ≥1 time in three years |
| Original articles in English | ≥1 article in three years | No requirements |
| Gastric cancer surgery | ≥60 cases in three years | ≥30 cases in three years |
| Endoscopic resection of gastric cancer | ≥60 cases in three years | ≥30 cases in three years |
| Gastric cancer chemotherapy | ≥60 cases in three years | ≥30 cases in three years |
| Intraoperative rapid diagnosis (histology and cytology) | Available | No requirements |
| Autopsy | Available | No requirements |
| Additional fee for outpatient chemotherapy | No requirements | Must be obtained |

Supplementary table 2. Lists of facilities participated in this survey.

Ageo Central General Hospital

Aomori City Hospital

Baba Memorial Hospital

Chiba Cancer Center

Chiba University Hospital

Dokkyo Medical University Hospital

Dokkyo Medical University Saitama Medical Center

Ebina General Hospital

Ehime Prefectural Central Hospital

Fuji City General Hospital

Fujisawa City Hospital

Fujita Health University Hospital

Fujita Health University Okazaki Medical Center

Fukui-ken Saiseikai Hospital

Fukuoka University Chikushi Hospital

Funabashi Municipal Medical Center

Gifu University Hospital

Gunma University Hospital

Hakodate Goryoukaku Hospital

Hamamatsu University Hospital

Higashiosaka City Medical Center

Hirosaki University Hospital

Hiroshima Citizens Hospital

Hiroshima City North Medical Center Asa Citizens Hospital

Hiroshima Memorial Hospital

Hiroshima Prefectural Hospital

Hokkaido Gastroenterology Hospital

Hokkaido University Hospital

Hyogo Cancer Center

Hyogo Medical University Hospital

Iizuka Hospital

Imamura General Hospital

Institute of Science Tokyo Hospital

Ishikawa Prefectural Central Hospital

Itami City Hospital

Iwate Medical University Hospital

Izumi City General Hospital

JA Hiroshima General Hospital

JA Onomichi General Hospital

Japan Community Healthcare Organization Osaka Hospital

Japanese Red Cross Fukuoka Hospital

Japanese Red Cross Kumamoto Hospital

Japanese Red Cross Kyoto Daiichi Hospital

Japanese Red Cross Kyoto Daini Hospital

Japanese Red Cross Musashino Hospital

Japanese Red Cross Osaka Hospital

Japanese Red Cross Tokushima Hospital

Japanese Red Cross Yamaguchi Hospital

Juntendo University Hospital

Kagawa Prefectural Central Hospital

Kagoshima University Hospital

Kaizuka City Hospital

Kanagawa Cancer Center

Kanazawa University Hospital

Kansai Medical University Medical Center

Keiyukai Sapporo Hospital

Kindai University Hospital

Kindai University Nara Hospital

Kiryu Kosei General Hospital

Kishiwada Tokushukai Hospital

Kitasato University Hospital

Kobe City Medical Center General Hospital

Kobe University Hospital

Komaki City Hospital

Konan Kosei Hospital

Kurume University Hospital

Kyoto University Hospital

Matsushita Memorial Hospital

Matsuyama Shimin Hospital

Mie University Hospital

Mimihara General Hospital

Miyagi Cancer Center

Nagaoka Chuo General Hospital

Nagoya City University Hospital

Nagoya University Hospital

Nara City Hospital

Nara Medical University Hospital

Nara Prefecture General Medical Center

Narita Memorial Hospital

National Cancer Center Hospital

National Hospital Organization Hokkaido Cancer Center

National Hospital Organization Hokkaido Medical Center

National Hospital Organization Iwakuni Clinical Center

National Hospital Organization Kanmon Medical Center

National Hospital Organization Kyushu Cancer Center

National Hospital Organization Kyushu Medical Center

National Hospital Organization Nagoya Medical Center

National Hospital Organization Oita Medical Center

National Hospital Organization Sendai Medical Center

National Hospital Organization Utsunomiya National Hospital

National Hospital Organization Yokohama Medical Center

National Hospital Organization Yonago Medical Center

New Tokyo Hospital

Nihon University Hospital

Niigata Cancer Center Hospital

Niigata City General Hospital

Niigata University Medical and Dental Hospital

Nippon Medical School Chiba Hokusoh Hospital

Nippon Medical School Hospital

NTT Medical Center Tokyo

Obihiro Kosei Hospital

Ogaki Municipal Hospital

Okayama City Hospital

Okayama Rosai Hospital

Okayama University Hospital

Okazaki City Hospital

Ome Medical Center

Osaka General Medical Center

Osaka Kaisei Hospital

Osaka Medical and Pharmaceutical University Hospital

Saga University Hospital

Saiseikai Nakatsu Hospital

Saiseikai Sendai Hospital

Saiseikai Shiga Hospital

Saiseikai Utsunomiya Hospital

Saiseikai Yokohamashi Nanbu Hospital

Saitama Medical University International Medical Center

Sakai City Medical Center

Seirei Hamamatsu General Hospital

Shiga University of Medical Science Hospital

Shimada General Medical Center

Shimane University Hospital

Shizuoka General Hospital

Showa General Hospital

Showa University Hospital

St.Marianna University Hospital

The Jikei University Hospital

Toho University Omori Medical Center

Tohoku Medical and Pharmaceutical University Hospital

Tokushima University Hospital

Tokyo Dental College Ichikawa General Hospital

Tokyo Medical University Ibaraki Medical Center

Tokyo Metropolitan Bokutoh Hospital

Tokyo Metropolitan Cancer and Infectious Diseases Center Komagome Hospital

Tokyo Shinagawa Hospital

Tokyo Women's Medical University Hospital

Toranomon Hospital

Toranomon Hospital Kajigaya

Tosei General Hospital

Tottori Prefectural Central Hospital

Tottori University Hospital

Toyonaka Municipal Hospital

Toyota Memorial Hospital

Tsuchiura Kyodo General Hospital

Tsuyama Chuo Hospital

University of Fukui Hospital

University of Miyazaki Hospital

University of Tokyo Hospital

University of Tsukuba Hospital

University of Yamanashi Hospital

Uonuma Kikan Hospital

Yamagata Prefectural Central Hospital

Yamagata Saisei Hospital

Yao Municipal Hospital

Yokohama Municipal Citizen's Hospital
